# Effects of multidisciplinary teamwork in non-hospital settings on healthcare and patients with chronic conditions: a systematic review and meta-analysis

**DOI:** 10.1186/s12875-025-02814-0

**Published:** 2025-04-15

**Authors:** Yanli Shi, Hongmin Li, Beibei Yuan, Xin Wang

**Affiliations:** 1 School of Public Health, Sun Yat-Sen University, No.74, the 2nd Zhongshan Road, Guangzhou, Guangdong Province 510080 China; 2 School of Public Health, Jining Medical University, Jining, China; 3 China Center for Health Development Studies Peking University, Beijing, China

**Keywords:** Multidisciplinary teamwork, Effects, Chronic conditions, Systematic review, Meta-analysis

## Abstract

**Background:**

There is evidence that multidisciplinary teams can improve health outcomes for patients with chronic conditions, enhance the quality and coordination of care, and promote teamwork among staff in hospital settings. However, their effectiveness in non-hospital settings remains unclear. Therefore, we conducted a systematic review and meta-analysis to assess the effects of multidisciplinary teams on patients with chronic conditions, health professionals, and healthcare in non-hospital settings.

**Methods:**

We searched PubMed, Web of Science, Embase, EconLit, OpenGrey, China National Knowledge Infrastructure (CNKI), and WanFang for randomised controlled trials published before March 2025. Narrative syntheses were used to synthesise the characteristics of multidisciplinary teams, interventions, and effects. Data were statistically pooled using both random-effects and fixed-effects meta-analyses to synthesize the outcomes. The methodological quality of the included studies was assessed using Cochrane’s risk of bias tool.

**Results:**

Thirty-nine studies were analyzed, with a total of 8186 participants. Nurses, general practitioners, and specialists were the most common members of the multidisciplinary teams. Staffing models, shared care and role expansion or task shifting are the most common multidisciplinary teamwork interventions. Narrative syntheses revealed improvements in self-management, self-efficiency, satisfaction, health behaviours, and knowledge. A meta-analysis found a significant reduction in hospitalisation days for patients with chronic obstructive pulmonary disease (MD=-0.66, 95% CI -1.05 to -0.26, I^2^ = 0%) and significant improvement in quality of life for patients with chronic heart failure (MD=-4.63, 95% CI: -8.67 to -0.60, I^2^ = 0%). There is no consistent evidence of other indicators of this effect.

**Conclusions:**

Multidisciplinary teamwork can improve patient-reported outcomes for patients with chronic conditions in non-hospital settings, but the effects on clinical outcomes, health utilisation, and costs are not evident.

**Trial registration:**

The study protocol was registered with PROSPERO on January 21, 2019, with the registration number CRD42019121109.

**Supplementary Information:**

The online version contains supplementary material available at 10.1186/s12875-025-02814-0.

## Introduction

The epidemic of chronic conditions and multimorbidity is a challenging health problem worldwide, as it poses a significant burden for individuals, families, and communities. According to World Health Statistics 2023, four major non-communicable diseases (cardiovascular disease, cancer, chronic respiratory disease, and diabetes) collectively led to the deaths of 33.3 million people in 2019, an increase of 28% compared with 2000 [1]. The overall prevalence of multimorbidity among adults was 37.2% globally [2]. Additionally, the prevalence of other chronic conditions also increased dramatically, such as mental disorders and persistent communicable diseases. Patients with chronic conditions, especially those with multimorbidities, have a higher demand for continuous and coordinated healthcare from different health professionals [3]. 

Inter-professional collaboration among health professionals is advocated internationally as a means of addressing the increasingly complex health needs of patients with chronic conditions [4]. Multidisciplinary teamwork, a model of inter-professional collaboration, is a dynamic process involving two or more healthcare professionals with complementary backgrounds and skills exercising efforts to provide care for patients [5]. Multidisciplinary teamwork, especially teamwork with health professionals in communities, has been implemented in many countries, such as the Inter-professional Team in the Netherlands, the Integrated Primary Care Team and the Family Health Team in Canada [6]. Interdisciplinary collaboration among healthcare professionals in a multidisciplinary team strengthens actions to prevent, diagnose, treat, and manage patients with chronic conditions and promote their health and well-being in a timely and continuous manner [7]. Theoretically, multidisciplinary teams can provide a more comprehensive understanding of patient needs. Based on this understanding, they can develop more comprehensive and integrated intervention strategies, provide better patient management, and, therefore, improve chronic disease diagnosis and management performance.

The growing practice of multidisciplinary teamwork has drawn attention to its effects on the healthcare and outcomes of patients with chronic conditions. There is a lack of conclusive evidence on the effects of multidisciplinary teamwork on the quality of care, healthcare utilisation and costs, and patient satisfaction in chronic patients in general. Comprehensive geriatric assessment by multidisciplinary teams for community-dwelled older adults increased the chance of living at home, reduced mortality, and improved cognitive function [8]. Community-based, Flexible, Assertive Treatment by multidisciplinary teams could reduce risks of cardiovascular disease [9], and had a positive impact on improving the physical activity level for patients with severe mental disorders [10]. In addition, there is some evidence of multidisciplinary teamwork on better health outcomes for patients with a single chronic condition, such as chronic lower back pain, hypertension, or diabetes or specific population groups, such as older adults [11–14]. However, there was also some evidence that multidisciplinary teamwork had no significant effect on the quality of life and survival of patients with diabetic foot or led to a longer diagnosis-to-treatment interval in cancer patients [15, 16]. 

Regarding the settings of multidisciplinary teamwork, current evidence from systematic reviews has shown that multidisciplinary teamwork in hospitals always improves the quality and coordination of care, shortens hospitalisation days, and improves job satisfaction and teamwork for healthcare staff [17, 18]. However, evidence about the effect of multidisciplinary teamwork in non-hospital settings (primary care, community, or family settings) is vague. Pooled quantified effects of multidisciplinary teamwork conducted in non-hospital settings were limited because of the substantial heterogeneity between studies [19]. Additionally, as the existing studies focus on developed countries, there is extremely limited evidence for policymakers in low-and middle-income countries [20]. Therefore, existing evidence is inconclusive to inform decision-makers in all countries of the potential effects of moving towards multidisciplinary teamwork on chronic patients and health care in non-hospital settings.

This systematic review aimed to provide evidence on the effects of multidisciplinary teamwork on healthcare and the outcomes of patients with chronic conditions in non-hospital settings. The current study can contribute to providing up-to-date evidence for the effects of multidisciplinary teams on patients with chronic diseases and effective interventions provided by teams in developed and developing countries, especially in community and family settings.

## Methods

We conducted a systematic review with narrative synthesis and meta-analysis, qualitatively extracting the characteristics and effects of interventions in multidisciplinary teams and quantitatively assessing the effects of these interventions on patients, health professionals, and healthcare. Owing to the heterogeneity in the interventions, a subgroup meta-analysis was conducted according to chronic conditions. Our review was registered with PROSPERO (CRD42019121109). It was reported accordingly with the Preferred Reporting Items for Systematic Reviews and Meta-Analyses (PRISMA) guideline [21]. 

### Search strategy and inclusion criteria

#### Data source and search strategy

We searched PubMed, Embase, Web of Science, EconLit, OpenGrey, CNKI and WanFang for studies published before March 2025. Additionally, we manually searched the reference lists of all included studies and review articles for additional references. The terms multidisciplinary team, chronic conditions, and effects were combined using the same search strategy for each database. The complete search strategies for all databases are provided in Supplementary Table S1 to S7.

#### Inclusion criteria and exclusion criteria

Studies were included in the review if they met all the following criteria: (a) the patients were diagnosed with one or more chronic conditions, (b) the intervention was provided by multidisciplinary teams comprising members from multiple disciplines, (c) the study settings were not in hospitals, (d) the study design was randomised controlled trials (RCTs), and (e) the study measured the effects from the perspectives of patients or healthcare providers.

Studies were excluded if they met any of the following criteria: (a) the study was published in a language other than English or Chinese, (b) the full text was not available, (c) the study was a sub-study derived from a larger study with identical inclusion criteria and intervention measures, (d) healthcare for both the intervention group and the control group was provided by a multidisciplinary team.

To be included in the meta-analysis, studies must simultaneously meet the following three criteria: (a) focus on the same disease, (b) evaluate the same outcome measures, and (c) have available data.

### Screening and extraction of data

#### Screening of studies

**Fig. 1:**
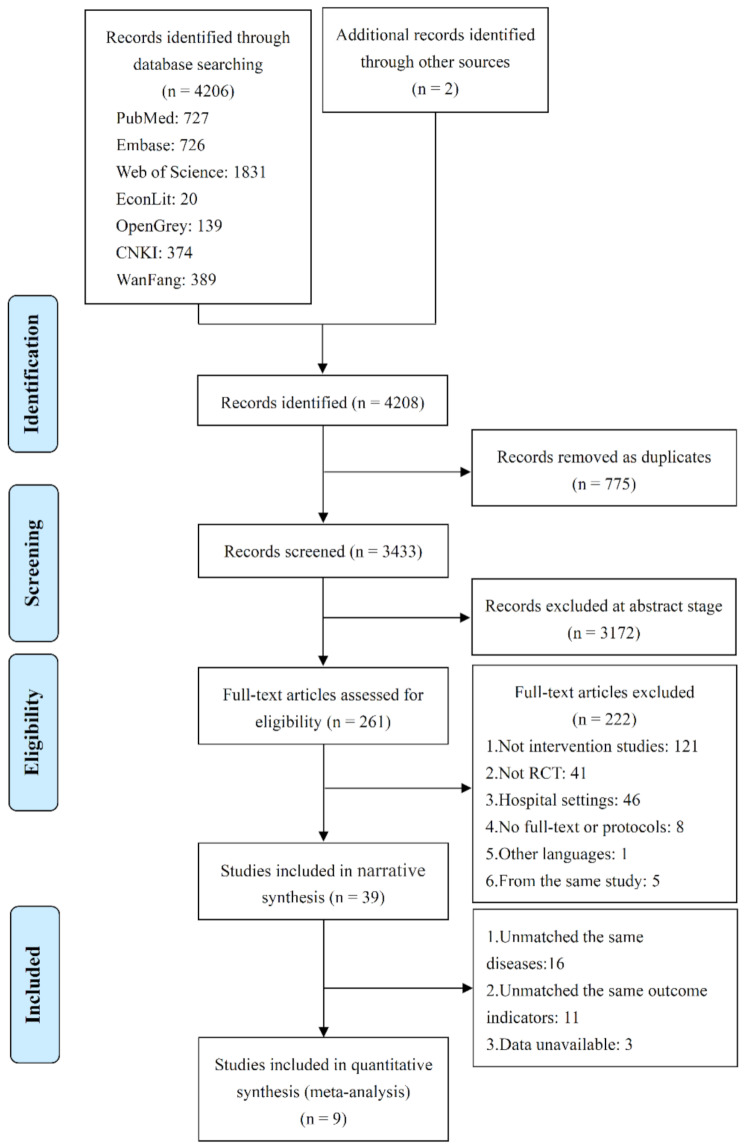
Flow-chart of study selection according to PRISMA

Two researchers (YS and HL) independently scanned the titles and abstracts of all identified studies in EndNote X9 software. They excluded those that were not relevant to the effectiveness of multidisciplinary teams for chronic conditions in non-hospital settings. The full text of all remaining studies was assessed for eligibility. If there was a disagreement, a third researcher (XW) was consulted. The identified studies were screened between July and September 2023, and updated in March 2025. The screening process and results are reported in Fig. 1. A consensus was reached among the three researchers of the included studies.

#### Data extraction

Two researchers extracted the data from the included studies in a dependent manner in a predetermined standardised spreadsheet form. The spreadsheet mainly included three parts: (a) characteristics of the studies, location, publication year, target population/patients (b) characteristics of the multidisciplinary teams and interventions conducted in the teams, and (c) effects of interventions, such as patients’ clinical outcomes, health utilisation, and costs.

### Quality assessment

Two researchers (HL and XW) independently assessed the risk of bias in the included studies using the quality criteria for randomised controlled trials (Cochrane Effective Practice and Organization of Care Review Group) [22]. The criteria included concealment of allocation, follow-up of professionals, follow-up of patients or episodes of care, blinded assessment of primary outcomes, baseline measurements, reliable primary outcome measures, and protection against contamination. Disagreements regarding quality ratings were resolved through discussions. No studies were excluded because of the risk of bias.

### Data synthesis and analysis

Narrative syntheses were used to synthesise the characteristics, interventions, and effects of the multidisciplinary teams. The characteristics of multidisciplinary teams reflect the composition of the teams’ disciplines. Two researchers (YS and XW) categorised the interventions in multidisciplinary teams of included studies using the Cochrane Effective Practice and Organization of Care (EPOC) taxonomy of health system interventions [23]. The EPOC taxonomy covers four topics, and interventions in included studies fit into the category of Delivery Arrangement, especially the subcategory Coordination of Care and Management of Care Processes. The effects of the intervention were categorised into health outcomes, utilisation of health services, and costs.

If two or more studies targeted the same chronic conditions and reported the same outcomes with extractable data, a meta-analysis was conducted to quantitatively assess the effect. We recorded the mean difference (MD) or standardised mean difference (SMD) with a 95% CI for continuous outcomes. We used *Ch*^2^ and *I*^2^ to assess the statistical heterogeneity. If the heterogeneity exceeded 50%, calculations were performed using a random-effects model in the RevMan software, whereas the fixed-effects model was utilised if it was below 50%. The results are reported as pooled effects and forest plots. If the data were allowed, a funnel plot was used to show publication bias.

### Role of the funding source

The funding source had no role in the design, execution, analyses, interpretation of the data, or decision to submit results.

## Results

### Search results

A total of 4208 records were pooled, of which 775 (18.42%) were duplicates. The titles and abstracts of 3433 (81.58%) non-duplicate records were screened, of which 3172 (75.38%) were excluded, and 261 (6.20%) remained. Of the 261 potentially eligible records, 39 (0.93%), with a total of 8186 participants, were included in our study. Figure 1 shows the search and selection of studies based on PRISMA flow-charts.

### Study characteristics

Table 1 presents the characteristics of the included 39 RCT studies. More than 70% of the studies were published between 2004 and 2020 (*n* = 30, 76.92%). More than two-third of the studies were conducted in Asia and North America (*n* = 28, 71.79%). Regarding settings of practice, most studies were conducted in community settings (*n* = 24, 63.10%), followed by the clinic settings (*n* = 8, 20.51%). The mean age of the participants in 30 studies (76.92%) was over 60 years. 27 of the 39 studies targeted patients with one chronic disease, including coronary heart disease (*n* = 5, 12.82%), chronic heart failure and chronic kidney disease (*n* = 4, 10.26%). Eight studies reported comorbidity of patients.

**Table 1:** Description of included studies

Study ID	Author, year	Country	Diseases (Comorbidities)	Participants	
				Number	Age
1	Kong et al., 2015	China	CHD	140	65.47 ± 8.12
2	Sun et al., 2016	China	CHD	151	63.84 ± 10.43
3	Xu et al., 2019	China	CHD	236	65.46 ± 6.0
4	Chen et al., 2019	China	CHD	80	64.92 ± 6.20
5	Jiang et al., 2020	China	CHD	144	61.03 ± 5.27
6	Austin et al., 2009	The UK	CHF	200	71.85 ± 6.54
7	Stewart., 2015	Australia	CHF	611	66± 11
8	Pan et al., 2016	China	CHF	90	72.98 ± 8.81
9	Bekelman et al., 2018	The US	CHF (HTN, DM, COPD, OSA, AF, MI, depression)	314	65.5 ± 11.4
10	Barrett et al., 2011	Canada	CKD	474	67
11	Gu et al., 2019	China	CKD	420	61.00 ± 13.45
12	Saudan et al., 2020	Switzerland	CKD (DM)	242	70 ± 8
13	Jhamb et al., 2023	The US	CKD (Fatigue, pain, depression)	160	58 ± 14
14	Hoogendoorn et al., 2010	Netherlands	COPD	199	66.47 ± 9.08
15	Hernandez et al., 2015	Spain	COPD	155	74.08 ± 8.59
16	Zhang et al., 2019	China	COPD	120	57.20 ± 5.10
17	Towfighi et al., 2021	The US	Stroke, TIA	487	57.1 ± 8.9
18	Markle-Reid et al., 2023	Canada	Stroke (CVD, DM, pain, musculoskeletal disorders, gastrointestinal disorders, kidney diseases, hearing disorders, vision disorders)	90	69.83 ± 9.41
19	Wu et al., 2023	China	Stroke	100	60.78 ± 10.84
20	Siaw et al., 2017	Singapore	T2DM (HTN, IHD, CKD, dyslipidaemia)	411	59.60 ± 8.12
21	Zhou et al., 2020	China	T2DM	62	54.70 ± 4.90
22	Walrabenstein et al., 2023	Netherlands	Hip OA, Knee OA	64	63
23	Tan et al., 2024	Singapore	Knee OA	110	66.11 ± 8.09
24	Chen et al., 2018	China	HTN	200	62.20 ± 8.85
25	Martin et al., 2014	Spain	FM (COPD, HTN, DM, RA, hypothyroidism)	153	50.12 ± 9.07
26	Sally et al., 2004	Australia	AF (COPD, HTN, IHD, DM)	152	73 ± 9
27	Preen et al., 2005	Australia	Chronic cardiorespiratory disease	189	75.1 ± 10.9
28	Pedersen et al., 2025	Denmark	Chronic hematological malignancies	94	64.4
29	Petronella et al., 2005	Netherlands	RA, AS, SLE, PsA, ReA, scleroderma	140	43.47
30	Pearson et al., 2006	Australia	Cardiac disease, respiratory disease, orthopedic condition, vascular disease	528	69.02 ± 11.51
31	Hogg et al., 2009	Canada	CAD, CHF, COPD, DM	241	71.20
32	Gray et al., 2010	Canada	CAD, CHF, COPD, DM	152	72.09
33	Bekelman et al., 2024	The US	COPD, CHF, ILD	306	68.9 ± 7.7
34	Radwany et al., 2014	The US	CHF, COPD, DM, CKD, CAD, ALS, PD, cirrhosis	80	69.15
35	Fisher et al., 2020	Canada	RA, DM, CVD, thyroid, gastrointestinal, renal and genitourinary	59	NA
36	Fortin et al., 2021	Canada	CVD, COPD, DM, asthma, hyperlipidemia	284	60.80 ± 10.45
37	Jiang et al., 2017	China	HTN, CHD, DM, hyperlipidemia	200	73.65 ± 5.61
38	Katon et al., 2010	The US	DM, CHD (Depression)	212	56.90 ± 11.34
39	Mildred et al., 2010	The US	Depression (HTN, HC, DM, hypothyroidism, asthma, chronic bronchitis, arthritis, heart disease, stroke)	136	55.23 ± 12.63

Notes: CHD: coronary heart disease, CHF: chronic heart failure, HTN: hypertension, DM: diabetes mellitus, COPD: chronic obstructive pulmonary disease, OSA: obstructive sleep apnea, AF: atrial fibrillation, MI: myocardial infarction, CKD: chronic kidney disease, TIA: transient ischemic attack, CVD: cardiovascular disease, T2DM: type 2 diabetes mellitus, IHD: ischaemic heart disease, Hip/Knee OA: hip/knee osteoarthritis, FM: fibromyalgia, RA: rheumatoid arthritis, AS: ankylosing spondylitis, SLE: systemic lupus erythematosus, PsA: psoriatic arthritis, ReA: reactive arthritis, CAD: coronary artery disease, ILD: interstitial lung disease, ALS: amyotrophic lateral sclerosis, PD: parkinson’s disease, HC: high cholesterol, NA: not available

### Risk of bias assessment

**Fig. 2:**
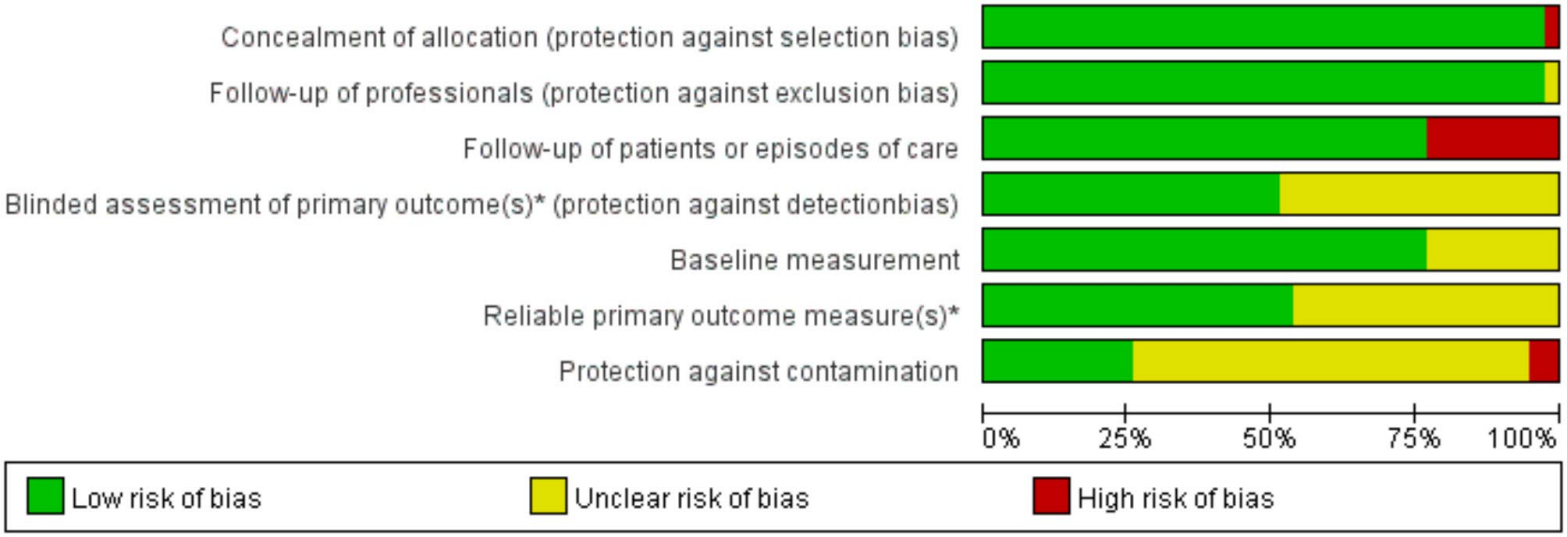
Risk of bias assessment

The quality assessment of the included studies is shown in Fig. 2. None of the studies met all of the quality criteria for low risk. All studies were rated as having high risk or unclear risk in at least one domain. We judged 27 studies to have unclear risk of bias [24–50]. Twelve studies had a high risk of bias, and the most common biases were attrition biases(*n* = 9, 23.08%), contamination (*n* = 2, 5.13%) and selection biases (*n* = 1, 2.56%) [51–62].

### Characteristics of multidisciplinary teams and interventions

As shown in Table 2, there were two to eight healthcare professionals among the multidisciplinary teams. Nurses were the most common member (*n* = 33, 84.62%), followed by general practitioners (*n* = 27, 69.23%), specialists (*n* = 25, 64.10%), and therapists (*n* = 15, 38.46%). Therapists are health professionals, such as physiotherapists, occupational therapists, speech therapists, and related specialists who deliver diagnostic, therapeutic and rehabilitation care. Equipped with advanced expertise in specialized clinical domains, they collaborate within interdisciplinary healthcare teams to optimize patient outcomes. Sixteen studies reported special roles, such as care manager or coordinator, in the multidisciplinary teams. The coordinators were mainly nurses (*n* = 7, 43.75%). According to EPOC taxonomy of interventions, interventions in the studies mainly focused on the category of Delivery Arrangements (as shown in Table 2). All studies included teams and coordination of care amongst different providers. Additionally, staffing models (*n* = 32, 82.05%) were the most common intervention of the studies, followed by share care (*n* = 26, 66.67%), role expansion or task shifting (*n* = 18, 46.15%), outreach services (*n* = 17, 43.59%), and self-management (*n* = 17, 43.59%). The duration of interventions ranged from 7 days to 24 months, and the follow-up lasted for 3 months to 60 months.

**Table 2:** Characteristics of multidisciplinary teams and interventions

Author, year	Setting	Team composition	Intervention description	Intervention duration	follow-up duration
Kong et al., 2015	Community	GP, specialist, nurse	Teams, group versus individual care, coordination of care amongst different provider, outreach services, self-management, staffing models	3 months	3 months
Sun et al., 2016	Community	Specialist, nurse^#^, dietitian, therapist, non-clinical coordinator	Disease management, shared decision-making, teams, group versus individual care, coordination of care amongst different provider, outreach services, site of service delivery, self-management, staffing models, health information systems	6 months	6 months
Xu et al., 2019	Community	GP, specialist, public health physicians, non-clinical coordinator	Care pathways, disease management, referral systems, shared care, teams, coordination of care amongst different provider, triage, outreach services, site of service delivery, staffing models, health information systems	NA	24 months
Chen et al., 2019	Community	GP, nurse, public health physicians	Referral systems, teams, coordination of care amongst different provider, self-management, staffing models	12 months	12 months
Jiang et al., 2020	Community	GP, nurse^#^, dietitian, therapist	Disease management, packages of care, referral systems, shared care, shared decision-making, teams, group versus individual care, coordination of care amongst different provider, triage, outreach services, site of service delivery, role expansion or task shifting, self-management, staffing models	6 months	6 months
Austin et al., 2009	Clinic	Specialist, nurse, dietitian, therapist	Packages of care, shared care, teams, coordination of care amongst different provider, site of service delivery, self-management, staffing models	6 months	60 months
Stewart et al., 2015	Home	GP, specialist, nurse^#^	Case management, shared care, teams, coordination of care amongst different provider, outreach services, site of service delivery, role expansion or task shifting, staffing models	NA	(51.0 ± 8.2) months
Pan et al., 2016	Community	GP, specialist, nurse, social worker, informal caregiver	Disease management, referral systems, shared care, teams, coordination of care amongst different provider, quality and safety systems, triage, outreach services, site of service delivery, staffing models	NA	12 months
Bekelman et al., 2018	Clinic	GP, specialist, nurse, social worker	Case management, shared care, shared decision-making, teams, coordination of care amongst different provider, outreach services, role expansion or task shifting, staffing models	6 months	6 months
Barrett et al., 2011	Community	GP, specialist, nurse^#^	Shared care, shared decision-making, teams, coordination of care amongst different provider, self-management, staffing models	24 months	24 months
Gu et al., 2019	Clinic	Specialist, nurse, dietitian, psychologist	Case management, Patient-initiated appointment systems, teams, group versus individual care, coordination of care amongst different provider, self-management, length of consultation, staffing models, health information systems, the use of information and communication technology	12 months	12 months
Saudan et al., 2020	Community	GP, specialist, nurse, dietitian	Teams, coordination of care amongst different provider, role expansion or task shifting, length of consultation, staffing models	NA	(51 ± 20) months
Jhamb et al., 2023	Clinic, Home	GP, specialist, therapist^#^, psychologist,	Case management, shared care, shared decision-making, teams, coordination of care amongst different provider, role expansion or task shifting, Telemedicine	3 months	12 months
Hoogendoorn et al., 2010	Community	Nurse, dietitian, therapist	Packages of care, teams, coordination of care amongst different provider, self-management, staffing models	NA	24 months
Hernandez et al., 2015	Community	GP, nurse, social worker	Packages of care, teams, coordination of care amongst different provider, outreach services, self-management, staffing models, the use of information and communication technology	NA	12 months
Zhang et al., 2019	Community	GP, specialist, nurse	Case management, communication between providers, continuity of care, packages of care, referral systems, shared care, teams, coordination of care amongst different provider, triage, outreach services, site of service delivery, staffing models, the use of information and communication technology	6 months	6 months
Towfighi et al., 2021	Community	GP, specialist, nurse, social worker	Continuity of care, disease management, packages of care, referral systems, shared care, shared decision-making, teams, coordination of care amongst different provider, outreach services, site of service delivery, role expansion or task shifting, self-management, staffing models, the use of information and communication technology	12 months	12 months
Markle-Reid et al., 2023	Community	Nurse, therapist^#^, social worker	Case management, packages of care, referral systems, shared care, shared decision-making, teams, coordination of care amongst different provider, role expansion or task shifting, self-management, length of consultation, health information systems	6 months	12 months
Wu et al., 2023	Community	Specialist, nurse, public health physicians^#^	Case management, continuity of care, teams, coordination of care amongst different provider, outreach services, length of consultation, the use of information and communication technology	6 months	6 months
Siaw et al., 2017	Primary care institution	GP, nurse, dietitian, pharmacist	Referral systems, teams, coordination of care amongst different provider, role expansion or task shifting, staffing models	6 months	6 months
Zhou et al., 2020	Community	GP, specialist, nurse, therapist	Case management, continuity of care, referral systems, shared care, teams, group versus individual care, coordination of care amongst different provider, site of service delivery, staffing models	12 months	12 months
Walrabenstein et al., 2023	Clinic	Dietitian, therapist	Packages of care, shared care, teams, group versus individual care, coordination of care amongst different provider, self-management, staffing models	4 months	4 months
Tan et al., 2024	Community	Specialist, dietitian, therapist, psychologist, social worker	Disease management, packages of care, shared care, teams, group versus individual care, coordination of care amongst different provider, triage, staffing models	3 months	12 months
Chen et al., 2018	Community	GP^#^, specialist, nurse, public health physicians	Case management, packages of care, teams, coordination of care amongst different provider, role expansion or task shifting, staffing models	12 months	12 months
Martin et al., 2014	Clinic	GP, therapist, psychologist	Packages of care, shared care, teams, group versus individual care, coordination of care amongst different provider, self-management, staffing models	6 weeks	12 months
Sally et al., 2004	Home	GP, specialist, nurse^#^, pharmacist	Shared care, teams, coordination of care amongst different provider, triage, outreach services, role expansion or task shifting, self-management, staffing models	6 months	60 months
Preen et al., 2005	Community	GP, specialist, nurse	Continuity of care, discharge planning, packages of care, shared care, shared decision-making, teams, coordination of care amongst different provider, site of service delivery, role expansion or task shifting, staffing models, the use of information and communication technology	7 days	7 days
Pedersen et al., 2025	Clinic	Specialist, nurse^#^, informal caregiver	Packages of care, teams, coordination of care amongst different provider, role expansion or task shifting, the use of information and communication technology	12 months	12 months
Petronella et al., 2005	Clinic	GP, specialist, therapist, psychologist, social worker, non-clinical coordinator^#^	Teams, coordination of care amongst different provider, staffing models	1-3months	24 months
Pearson et al., 2006	Home	GP, nurse, pharmacist	Discharge planning, shared care, teams, coordination of care amongst different provider, outreach services, role expansion or task shifting, staffing models	6 months	90 months
Hogg et al., 2009	Community	GP, nurse, pharmacist	Disease management, shared care, shared decision-making, teams, coordination of care amongst different provider, outreach services, staffing models	NA	12–18 months
Gray et al., 2010	Primary care institution	GP, nurse, pharmacist	Disease management, shared care, teams, coordination of care amongst different provider, outreach services, site of service delivery, staffing models, smart home technologies	NA	12–18 months
Bekelman et al., 2024	Community	GP, specialist, nurse, social worker	Shared care, shared decision-making, teams, coordination of care amongst different provider, length of consultation, the use of information and communication technology	6 months	12 months
Radwany et al., 2014	Community	Specialist, nurse, dietitian, therapist, pharmacist, psychologist, social worker, non-clinical coordinator^#^	Care pathways, comprehensive geriatric assessment, continuity of care, disease management, packages of care, shared care, shared decision-making, teams, coordination of care amongst different provider, triage, outreach services, site of service delivery, role expansion or task shifting, self-management, staffing models	12 months	12 months
Fisher et al., 2020	Community	Nurse, therapist, social worker, non-clinical coordinator^#^	Case management, communication between providers, shared care, shared decision-making, teams, coordination of care amongst different provider, outreach services, role expansion or task shifting, self-management, staffing models, the use of information and communication technology	6 months	6 months
Fortin et al., 2021	Community	Nurse^#^, dietitian, therapist	Care pathways, case management, communication between providers, shared care, teams, coordination of care amongst different provider, triage, role expansion or task shifting, staffing models	4 months	4 months
Jiang et al., 2017	Community	GP^#^, nurse, public health physicians, therapist	Case management, packages of care, teams, coordination of care amongst different provider, role expansion or task shifting	3 months	3 months
Katon et al., 2010	Primary care institution	GP, specialist, nurse, psychologist	Case management, packages of care, shared care, shared decision-making, teams, coordination of care amongst different provider, quality and safety systems, site of service delivery, self-management, staffing models	12 months	12 months
Mildred et al., 2010	Clinic	GP, specialist, nurse, psychologist^#^	Case management, shared care, shared decision-making, teams, coordination of care amongst different provider, triage, role expansion or task shifting	6 months	6 months

Notes: GP: general practitioner, ^#^Coordinator/Care manager, NA: not available

### Effects of interventions in multidisciplinary teams

As shown in Table 3, although targeted at different diseases, the included studies reported the following three outcomes: (a) health outcomes (*n* = 37, 94.87%), (b) utilization of health services (*n* = 17, 43.59%), and (c) costs (*n* = 8, 20.51%).

**Table 3:** Characteristics of outcomes

Study ID	Author, year	Health outcomes		Utilisation of health services	Costs
		Patient clinical or mental health outcomes	Patient-reported outcome measures		
1	Kong et al., 2015	**-**	Self-management*, self-efficacy*, quality of life*	**-**	**-**
2	Sun et al., 2016	**-**	Self-management*, self-efficacy*	**-**	**-**
3	Xu et al., 2019	Adverse event rates*	Self-efficacy*, quality of life*, knowledge*	**-**	**-**
4	Chen et al., 2019	-	Self-efficacy*, health behaviours*	**-**	**-**
5	Jiang et al., 2020		Self-management*, self-efficacy*, quality of life*	Visits*, re-admission rates*	**-**
6	Austin et al., 2009	Glycaemic*, triglycerides, depression	**-**	**-**	**-**
7	Stewart., 2015	Mortality	**-**	Hospitalisation days*, hospitalisation rate	**-**
8	Pan et al., 2016	**-**	Quality of life*	hospitalisation rate*, medications	**-**
9	Bekelman et al., 2018	Mortality, depression*	Symptom	hospitalisation rate	**-**
10	Barrett et al., 2011	Blood pressure	**-**	Medications	**-**
11	Gu et al., 2019	Glycaemic, blood pressure*, SCr*, eGFR*	Self-management*	**-**	**-**
12	Saudan et al., 2020	Mortality, SCr, eGFR	Quality of life	re-admission rates	**-**
13	Jhamb et al., 2023	Adverse events rate, depression*	Symptom*	**-**	**-**
14	Hoogendoorn et al., 2010	**-**	**-**	Hospitalisation days	Total hospital costs
15	Hernandez et al., 2015	Mortality*, depression*	Self-management*, quality of life*	Visits*, hospitalisation rate	**-**
16	Zhang et al., 2019	**-**	Self-efficacy*	Hospitalisation days*	Medical costs*
17	Towfighi et al., 2021	Glycaemic, blood pressure	**-**	**-**	**-**
18	Markle-Reid et al., 2023	**-**	Self-management*	re-admission rates	Total hospital costs
19	Wu et al., 2023	**-**	Self-management*, quality of life*, self-efficacy*	**-**	**-**
20	Siaw et al., 2017	Glycaemic*, blood pressure, triglycerides	Satisfaction*	**-**	Medical costs*
21	Zhou et al., 2020	Glycaemic*	Satisfaction*	Appointment rate of outpatient*	**-**
22	Walrabenstein et al., 2023	Glycaemic*, blood pressure, triglycerides	Symptom*	**-**	**-**
23	Tan et al., 2024	**-**	Knee injury and osteoarthritis outcome Score	**-**	Total hospital costs
24	Chen et al., 2018	Glycaemic*, blood pressure*, triglycerides*	Health behaviours*, knowledge*	**-**	**-**
25	Martin et al., 2014	**-**	Symptom*	**-**	**-**
26	Sally et al., 2004	Mortality*	**-**	Hospitalisation days*, re-admission rates*	**-**
27	Preen et al., 2005	**-**	Quality of life, satisfaction*	Hospitalisation days	**-**
28	Pedersen et al., 2025	**-**	Quality of life*, symptom*	**-**	**-**
29	Petronella et al., 2005	Depression*	Quality of life*	**-**	**-**
30	Pearson et al., 2006	Mortality	**-**	re-admission rates*	Unplanned hospitalisation costs*, total hospital costs*
31	Hogg et al., 2009	Glycaemic, blood pressure	Quality of life	Visits, hospitalisation rate	**-**
32	Gray et al., 2010	**-**	**-**	**-**	Program costs, cost-effectiveness
33	Bekelman et al., 2024	**-**	Quality of life*, symptom*	**-**	**-**
34	Radwany et al., 2014	**-**	Quality of life, symptom	Visits*	**-**
35	Fisher et al., 2020	Depression	Self-efficacy, quality of life	Visits, hospitalisation rate	Home care and outpatient service costs
36	Fortin et al., 2021	**-**	Self-management, self-efficacy*, quality of life, health behaviours*	**-**	**-**
37	Jiang et al., 2017	**-**	Quality of life*, satisfaction*	**-**	**-**
38	Katon et al., 2010	Adverse event rates, glycaemic*, blood pressure*, depression*	Quality of life*, satisfaction*	**-**	**-**
39	Mildred et al., 2010	Depression*	**-**	**-**	**-**

Notes: SCr: serum creatinine, eGFR: estimated glomerular filtration rate, Minus sign: not measured, * Outcome was statistically significant

- Health outcomes

Thirty-seven studies reported health outcomes. We then divided the health outcomes into patient clinical or mental health outcomes and patient-reported outcome measure categories based on whether the outcomes were self-reported.

- Patient clinical or mental health outcomes

Twenty-one studies reported clinical outcomes, including mortality, adverse event rate, glycaemic status, blood pressure, triglycerides, serum creatinine (SCr), estimated glomerular filtration rate (eGFR), and depression scores. Six studies [25–27, 31, 43, 60] reported mortality rates. Two studies [26, 27] reported that patient mortality was reduced in the intervention group, whereas four studies [25, 31, 43, 60] reported no significant intergroup differences. Two studies [29, 35] reported adverse event rates, and only one study [35] found the incidence in the intervention group was lower than that in the control group.

Nine studies [29, 32, 40, 41, 44, 45, 51, 56, 59] reported glycaemic. Six studies [29, 32, 41, 44, 51, 59] found that the HbA1c reduction in the intervention group was greater than that in the control group, and three studies [40, 45, 56] showed no significant differences between the two groups. Three studies [32, 41, 44] reported fasting blood glucose levels. Two studies [32, 41] showed a bigger fasting blood glucose reduction in the intervention group than in the control group and one study [44] showed no significant difference between the two groups. Eight studies [24, 29, 40, 41, 44, 45, 56, 59] reported blood pressure. Three studies [29, 40, 41] found that compared to the control group, patients in the intervention group showed greater improvement in blood pressure levels. Five studies [24, 44, 45, 56, 59] found there were no statistically significant intergroup differences between the two groups. Four studies [41, 44, 51, 59] reported triglycerides and three studies [44, 51, 59] showed no significant differences between the groups.

**Fig. 3:**
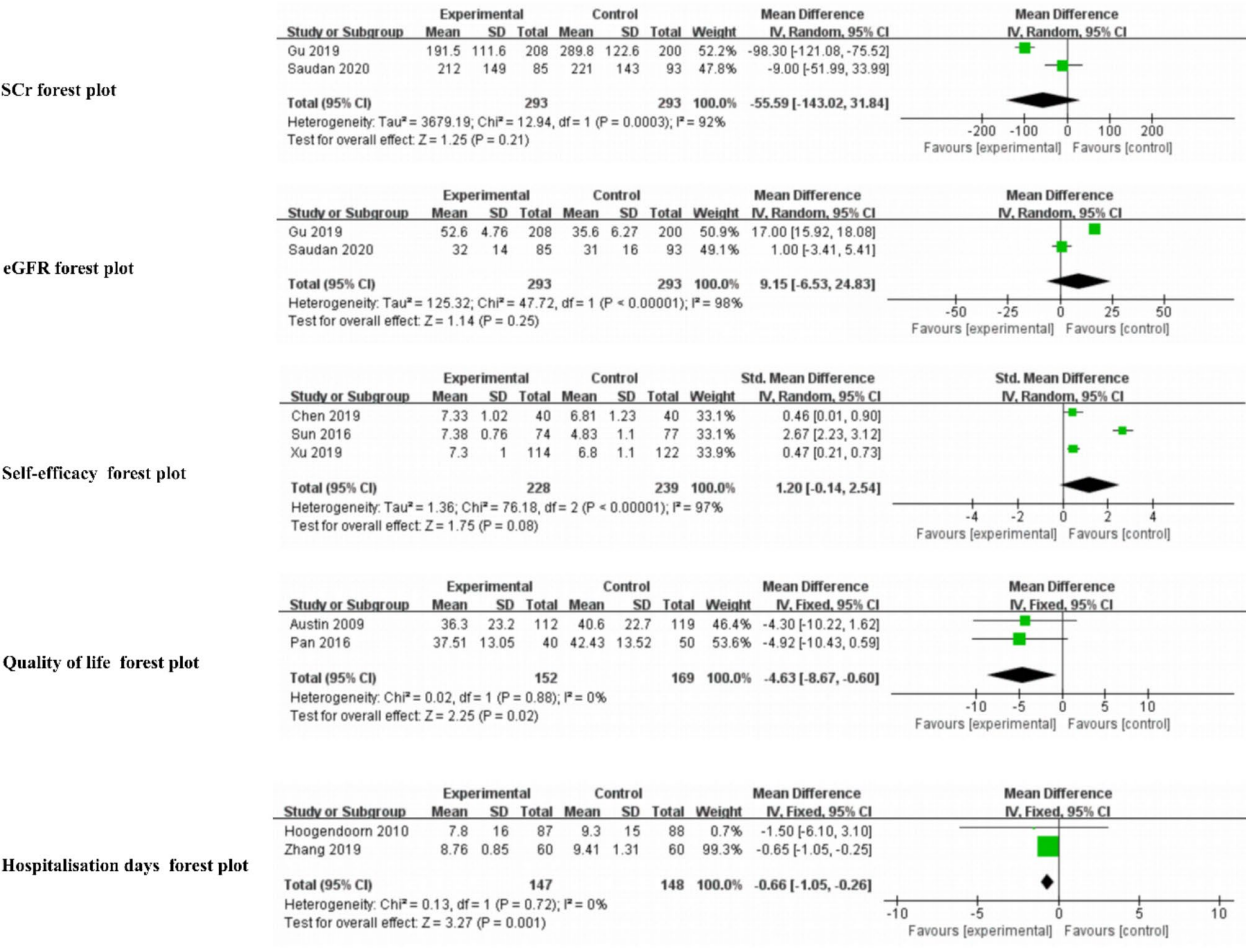
Forest plots of the outcome indicators

For chronic kidney disease, the meta-analysis of two studies [31, 41] showed no significant differences of SCr (MD=-55.59, 95% CI -143.02 to 31.84, *P* = 0.21, I^2^ = 92%) and eGFR (MD = 9.15, 95% CI -6.53 to 24.83, *P* = 0.25, I^2^ = 98%) between the two groups (Fig. 3).

Eight studies [25, 26, 29, 51–53, 63, 64] assessed depression scores, of which six studies [25, 26, 29, 52, 63, 64] reported a greater decrease in the intervention group than in the control group. Two studies [51, 53] showed no significant differences between the two groups.

b) Patient-reported outcome measures

Thirty studies reported patient-reported outcomes, including self-management scores, self-efficacy scores, quality of life, satisfaction, symptom scores, health behaviours, and knowledge. Eight studies [26, 28, 36, 38, 40, 48, 50, 54] reported self-management scores and nine studies [28, 34–36, 38, 42, 50, 53, 54] reported self-efficacy scores. And only one study [53, 54] indicated no statistically significant difference in self-management and self-efficacy between the two groups. The meta-analysis of three studies [35, 36, 43] of patients with coronary heart disease detected no significant differences between the two groups in self-efficacy scores (SMD = 1.20, 95% CI -0.14 to 2.54, *P* = 0.08, I^2^ = 97%), indicating a high degree of heterogeneity (Fig. 3).

Quality of life was reported in 17 studies, although different scales were used for measurement. Eleven studies [26, 28, 29, 35, 37–39, 46, 47, 50, 62] reported that patients in the intervention group reported greater improvements in their quality of life than patients in the control group. Six studies [33, 52–54, 56, 60] found that the quality of life did not differ between the two groups. A meta-analysis of the two studies [37, 46] for patients with chronic heart failure showed statistically significant differences in quality of life measured by the MLHFQ scale between the two groups (MD=-4.63, 95% CI -8.67 to -0.60, *P* = 0.02, I^2^ = 0%), indicating no heterogeneity (Fig. 3).

All five studies [29, 32, 39, 46, 59] that reported satisfaction with care consistently showed that patients in the intervention group were more satisfied with care than those in the control group. Symptom scores were reported in seven studies [25, 30, 33, 47, 61–63]. Five studies [30, 47, 61–63] reported a greater relief of symptoms in the intervention group than in the control group, whereas the other two studies [25, 33] found no differences between the two groups.

Five studies reported effects on health behaviours and knowledge. Three studies [41, 42, 54] reported improvements in health behaviours after the intervention, including tobacco smoking, physical activity, and healthy eating habits. Two studies [35, 41] reported that scores on the coronary heart disease and hypertension knowledge and cognition questionnaire were higher in the intervention group than in the control group.

(b) Utilisation of health services

Seventeen studies reported the effects on health service utilisation, including the appointment rate of outpatients, visits, hospitalisation days, hospital admission and re-admission, and medications. One study [32] reported that the appointment rate for outpatient services in the intervention group was higher than that in the control group. Five studies [26, 28, 33, 53, 56] reported visits. Three of them [26, 28, 33] showed the average number of physician visits per patient was lower in the intervention group. Six studies [25, 26, 37, 43, 53, 56] reported hospitalisation rate, one of them [37] found that the hospitalisation rate in the intervention group was lower than that in the control group. Five studies [27, 28, 31, 57, 60] reported re-admission rates, three of them [27, 28, 31] showed that patients in the intervention group had fewer re-admissions. There were no significant differences in the rate of medication use between the two studies [24, 37]. 

Hospitalisation days were reported in five studies [27, 34, 43, 46, 57]. three studies [27, 34, 43] reported shorter lengths of stay in the intervention group. A meta-analysis of patients with chronic obstructive pulmonary disease showed statistically significant differences between two groups in two studies [26, 34] (MD=-0.66, 95% CI -1.05 to -0.26, *P* = 0.001, I^2^ = 0%), with no heterogeneity (Fig. 3).

(c) Costs

Eight studies reported on costs, including medical costs, program costs, unplanned hospitalisation costs, total hospital costs, home care and outpatient service costs, and cost-effectiveness. Two studies [55, 58] reported program cost and cost-effectiveness analyses. One study [58] indicated that patients in the intervention group had lower total medical costs, with no reduction in their quality of life. One study [55] found that interventions might be more cost-effective when the program targets populations with poor care levels. Three studies [31, 34, 59] suggested that medical costs, unplanned hospitalisation costs, and total hospital costs in the intervention group were lower than those in the control group. One study [53] reported that home care and outpatient service costs were higher in the intervention group than in the control group because intervention costs were included in these costs.

## Discussion

To the best of our knowledge, this is the first systematic review to describe and evaluate multidisciplinary teamwork in non-hospital settings. We documented key characteristics of multidisciplinary teams and interventions in the literature, synthesised the effects of interventions, and quantified the effects of interventions on patients with specific conditions.

### Main findings and comparison with other studies

It shows that multidisciplinary teamwork is effective in improving most patient-reported outcomes (satisfaction, health behaviours, and knowledge). However, there is inconsistent evidence regarding the effects of improving clinical outcomes and reducing the utilisation of health services and costs.

Thirty-nine studies suggest that multidisciplinary teamwork is not only advocated but also implemented worldwide as a strategy to improve the management of chronic conditions. The consistent focus on community settings in the included studies provides empirical justification for prioritizing primary care systems for patients with chronic conditions. This finding resonates with Hickman’s review, which positions community-based care as critical for post-hospitalization populations. These studies, targeting chronic conditions and comorbidities, reflect the empirical consensus on the necessity for integrated care and inter-professional collaboration.

Variations in multidisciplinary team members resulted from the health demands of patients with different chronic conditions rather than differences among countries or regions. We found that one-third of the included studies reported key roles in teams, such as team leader, care coordinator, or manager, most of which were assigned to trained nurses. In the included studies, 85% of multidisciplinary teams had nurses who played a key role in the teams. In primary healthcare settings, nurses are more available and flexible than specialists for chronic conditions. They play an important role in team coordination and the management of chronic conditions. This is consistent with the findings in palliative care and residential long-term care by Dorja [65] and Kathrin [66], who found that nurses play a central role in care coordination.

Interventions in the teams focus on Delivery Arrangement in the EPOC taxonomy. Among the five subcategories of delivery arrangements, who provide care and how the healthcare workforce is managed are the most reported subcategories, rather than the Coordination of Care and Management of Care Process. In addition to teams and care coordination among different providers, role expansion and staffing models are among the most commonly reported interventions. This is also supported by Dubois and Singh [67], who found that the staff mix is a key element of integrated workforce management. Strategically ‘ensuring the right people, with the right skills, are in the right place at the right time’ enables optimal outcomes for patients, healthcare professionals and health systems (primarily costs and utilisation of health services). Notably, over half of the multidisciplinary teams promoted responsibility for healthcare or disease management to patients, respected patients’ autonomy, and maximally responded to each patient’s unique priorities. It is estimated that individuals and family carers perform more than 99% of daily care for chronic conditions [68]. Whereas, in our study, only two of the 39 studies involved informal caregivers in a multidisciplinary team. Over half of the multidisciplinary teams outreach the services of different providers in community primary care institutions. Outreach not only facilitates health services and improves patients’ experiences of people-centred care but also decreases travel costs and medical expenses.

This review showed that a multidisciplinary team is effective in improving most patient-reported outcomes. Compared with clinical outcomes, patient-reported outcomes are more important in demonstrating the impact of interventions on the outcomes most meaningful to patients and in informing policymaking from the perspective of patients and their families [69]. Moreover, patient-reported outcome measures were viewed as enablers of people-centred health care [70]. People-centeredness is one of the key aims of integrated multidisciplinary care. Therefore, it is reasonable to conclude that a multidisciplinary team is effective in improving most patient-reported outcomes. In the context of the primary healthcare delivery system, the sustained service and attention of a multidisciplinary team will enable chronic patients to improve their self-efficacy, quality of life, satisfaction, and health evaluation. From the perspective of the modern medical model, multidisciplinary teams are useful for enhancing overall health. However, evidence of its effects on clinical outcomes, utilisation of health services, and costs remains mixed. This is consistent with the findings of studies targeting some chronic conditions [71]. Hickman’s review demonstrated that multidisciplinary team interventions could reduce re-admission rates, mortality, and function decline in older patients [72]. Different healthcare settings and times of intervention partially resulted in different findings. Hickman’s review targeted acute care settings; the multidisciplinary team members were from different hospital units, and participants were inpatients with high disease severity facing a high risk of mortality and re-admission. Most teams in our review provided care in community settings for patients in a stable stage of chronic conditions with a low risk of mortality during follow-up. Therefore, the teamwork of multidisciplinary teams in acute and non-acute care settings is quite different, such as the effects on clinical outcomes and utilisation of care in the short term.

### Implication for further research and practice

The following four implications are provided for further research: First, the low rate of reporting of care coordinators or managers in the included studies reinforces the need for playing the role of qualified care coordinators in teams. As explained by Glouberman and Mintzberg [73], the health workforce delivers health care in different professional “worlds” in terms of setting (e.g. acute or chronic care) or service focus (e.g. cure or care). Qualified care coordinators have been proven to enhance multisectoral communication and promote professionalism with clear roles and tasks [65, 74]. Second, self-management interventions are core components of high-quality chronic disease care. The perspectives and behaviours of informal caregivers are as crucial as those of patients. Incorporating informal caregivers into multidisciplinary care teams is a promising approach to enhancing the appropriate perspectives and behaviours of informal caregivers [75]. Third, once the appropriate staff mix and skill management strategies are identified and the group of providers with a certain skill is assembled, we still need to organise the way in which the group cooperates and delivers care in practice. In addition to the multidisciplinary staff, multidisciplinary protocols, pathways, and team meetings are two distinct aspects of multidisciplinary teamwork. Therefore, it is necessary to develop multidisciplinary protocols for teams. Fourth, the effects of a multidisciplinary team may require a longer detection period, especially regarding clinical outcomes and health service utilisation of patients with chronic conditions. Regarding effect measurement, intermediate- and long-term outcomes are both suggested at different periods of intervention implementation.

### Limitations

However, this study has three limitations. First, there were some methodological deficiencies in the meta-analysis. The heterogeneity of the interventions and the relatively small number of studies and participants may have led to insufficient power to detect intervention effects. Second, we categorised the interventions based on the EPOC taxonomy and could not exclude the possibility of missing other interventions. Moreover, we were unable to estimate the specific effect size of the various EPOC interventions because each multidisciplinary team implemented several interventions. Third, there may be potential selection bias. Due to language limitations, our literature search was restricted to Chinese and English databases, excluding those in other languages. This may result in the overlook of evidence in other languages.

### Conclusion

Multidisciplinary teamwork can improve patient-reported outcomes such as satisfaction, health behaviour, and knowledge for patients with chronic conditions in community, clinic, and home settings. However, the effects on clinical outcomes, health utilisation, and costs are not evident in a relatively short period.

## Electronic supplementary material

Below is the link to the electronic supplementary material.

Supplementary Material 1

## Data Availability

The authors confrm that the data supporting the findings of this study areavailable within the article. The datasets analysed are available from the corresponding author, upon reasonable request.
