# Supplementary material for: Effects of multidisciplinary teamwork in non-hospital settings on healthcare and patients with chronic conditions: a systematic review and meta-analysis
Source: BMC Prim Care. 2025 Apr 15;26:110. doi: 10.1186/s12875-025-02814-0 (PMC11998469; doi:10.1186/s12875-025-02814-0)

### Supplementary Material

[Supplementary Table A.1. Search Strategy for PubMed 2](#_Toc176977743)

[Supplementary Table A.2. Search Strategy for Embase 4](#_Toc176977744)

[Supplementary Table A.3. Search Strategy for Web of Science（Web of Science Core Collection） 5](#_Toc176977745)

[Supplementary Table A.4. Search Strategy for Econlit 6](#_Toc176977746)

[Supplementary Table A.5. Search Strategy for OpenGrey 7](#_Toc176977747)

[Supplementary Table A.6. Search Strategy for WangFang 8](#_Toc176977748)

[Supplementary Table A.7. Search Strategy for CNKI 9](#_Toc176977749)

[Supplementary Table A.8. Search results 10](#_Toc176977750)

[Supplementary Fig. A.1. Risk of bias summary 11](#_Toc176977751)

### Supplementary Table A.1. Search Strategy for PubMed

| 1. "interdisciplinary" [Mesh] |
| --- |
| 1. "multidisciplinary" [Mesh] |
| 1. "interdisciplinary communication" [Mesh] |
| 1. "patient care team" [Mesh] |
| 1. "interdisciplinary" [Title/Abstract] |
| 1. "multidisciplinary" [Title/Abstract] |
| 1. "cross disciplinary" [Title/Abstract] |
| 1. "transdisciplinary" [Title/Abstract] |
| 1. "patient care team*" [Title/Abstract] |
| 1. "medical care team*" [Title/Abstract] |
| 1. "healthcare team*" [Title/Abstract] |
| 1. "health care team*" [Title/Abstract] |
| 1. "MDT" [Title/Abstract] |
| 1. "multiple providers" [Title/Abstract] |
| 1. "interprofessional" [Title/Abstract] |
| 1. "health team*" [Title/Abstract] |
| 1. "teamwork" [Title/Abstract] |
| #18 #1 or #2 or #3 or #4 or #5 or #6 or #7 or #8 or #9 or #10 or #11 or #12 or #13 or #14 or #15 or #16 or #17 |
| #19 "chronic disease" [Mesh] |
| #20  "chronic condition" [Mesh] |
| #21 "chronic illness" [Mesh] |
| #22  "noncommunicable disease" [Mesh] |
| #23 "noninfectious disease" [Mesh] |
| #24 "chronic disorder" [Mesh] |
| #25 "chronic patient" [Mesh] |
| #26 "chronic care" [Mesh] |
| #27 "chronic disease*" [Title/Abstract] |
| #28 "chronic condition*" [Title/Abstract] |
| #29 "chronic disorder*" [Title/Abstract] |
| #30 "chronic illness*" [Title/Abstract] |
| #31 "chronically ill" [Title/Abstract] |
| #32 "noncommunicable disease*" [Title/Abstract] |
| #33 "non-communicable disease*" [Title/Abstract] |
| #34 "noninfectious disease*" [Title/Abstract] |
| #35 "non-infectious disease*" [Title/Abstract] |
| #36 "chronic patient*" [Title/Abstract] |
| #37 "chronic care" [Title/Abstract] |
| #38 #19 or ~#37 |
| #39 "Clinical Trials Phase II as Topic"[Mesh] |
| #40 "Clinical Trials Phase III as Topic"[Mesh] |
| #41 "Clinical Trials Phase IV as Topic"[Mesh] |
| #42 "Controlled Clinical Trials as Topic"[Mesh] |
| #43  "Randomized Controlled Trials as Topic"[Mesh] |
| #44 "Intention to Treat Analysis"[Mesh] |
| #45 "Pragmatic Clinical Trials as Topic"[Mesh] |
| #46 "Clinical Trial Phase II"[Publication Type] |
| #47 "Clinical Trial Phase III"[Publication Type] |
| #48 "Clinical Trial Phase IV"[Publication Type] |
| #49 "Controlled Clinical Trial"[Publication Type] |
| #50 "Randomized Controlled Trial"[Publication Type] |
| #51 "Pragmatic Clinical Trial" [Publication Type] |
| #52 "Single-Blind Method"[Mesh] |
| #53 "Double-Blind Method"[Mesh] |
| #54 random*[Title/Abstract] |
| #55 blind*[Title/Abstract] |
| #56 single blind*[Title/Abstract] |
| #57 double blind*[Title/Abstract] |
| #58 triple blind*[Title/Abstract] |
| #59  "RCT"[Title/Abstract] |
| #60 #39 or ~#59 |
| #61 #18 AND #38 AND #60 |
| #62 Letter [PT] OR news [PT] OR comment [PT] OR editorial [PT] OR bibliography [PT] |
| #63 #61 NOT #62 |

### Supplementary Table A.2. Search Strategy for Embase

| #1 'interdisciplinary care'/exp OR 'multidisciplinary team'/exp OR 'multidisciplinary care'/exp OR 'interprofessional collaboration'/exp OR 'multidisciplinary approach'/exp OR 'multidisciplinary management'/exp |
| --- |
| #2  'interdisciplinary':ab,ti OR 'cross disciplinary':ab,ti OR 'transdisciplinary':ab,ti OR 'multidisciplinary':ab,ti OR 'interprofessional':ab,ti OR 'patient care team':ab,ti OR 'medical team':ab,ti OR 'healthcare team':ab,ti OR 'MDT':ab,ti OR 'multiple provider':ab,ti OR 'health team':ab,ti OR 'teamwork':ab,ti |
| #3 #1 OR #2 |
| #4 'chronic disease'/exp OR 'chronic diseases'/exp OR 'chronic patient'/exp OR 'chronic care model'/exp OR 'non communicable disease'/exp |
| #5 'chronic disease':ab,ti OR 'chronic diseases':ab,ti OR 'chronic illness':ab,ti OR 'chronic illnesses':ab,ti OR 'chronically ill':ab,ti OR 'chronic patient':ab,ti OR 'chronic patients':ab,ti OR 'non communicable disease':ab,ti OR 'non communicable diseases':ab,ti OR 'non-communicable disease':ab,ti OR 'non-communicable diseases':ab,ti OR 'noninfectious disease':ab,ti OR 'noninfectious diseases':ab,ti OR 'chronic condition':ab,ti OR 'chronic conditions':ab,ti OR 'chronic disorder':ab,ti OR 'chronic disorders':ab,ti OR 'chronic care':ab,ti |
| #6 #4 OR #5 |
| #7 'crossover procedure':de OR 'double-blind procedure':de OR 'randomized controlled trial':de OR  'single-blind procedure':de OR (random* OR  factorial* OR crossover* OR cross NEXT/1 over* OR placebo* OR doubl* NEAR/1 blind* OR singl* NEAR/1 blind* OR assign* OR allocat* OR volunteer*):de,ab,ti |
| #8 #3 AND #6 AND #7 |
| #9 'letter':it OR 'news':it OR 'comment':it OR 'editorial':it OR 'bibliography':it OR 'resource guides':it |
| #10 #8 NOT #9 |

### Supplementary Table A.3. Search Strategy for Web of Science（Web of Science Core Collection）

| #1 (((((((((((TS=(interdisciplinary)) OR TS=(multidisciplinary)) OR TS=("cross disciplinary")) OR TS=(transdisciplinary)) OR TS=("patient care team")) OR TS=("medical care team")) OR TS=("healthcare team")) OR TS=(MDT)) OR TS=("multiple providers")) OR TS=(interprofessional)) OR TS=("health team")) OR TS=(teamwork) |
| --- |
| #2 (((((((((((((((((((TS=("chronic disease")) OR TS=("chronic diseases")) OR TS=("chronic illness")) OR TS=("chronic illnesses")) OR TS=("chronically ill")) OR TS=("noncommunicable disease")) OR TS=("noncommunicable diseases")) OR TS=("non-communicable disease")) OR TS=("non-communicable diseases")) OR TS=("non communicable disease")) OR TS=("non communicable diseases")) OR TS=("noninfectious diseases")) OR TS=("noninfectious diseases")) OR TS=("chronic condition")) OR TS=("chronic conditions")) OR TS=("chronic disorder")) OR TS=("chronic disorders")) OR TS=("chronic patient")) OR TS=("chronic patients")) OR TS=("chronic care") |
| #3  ((((((((((TS=(clinical trial*)) OR TS=(research design)) OR TS=(comparative stud*)) OR TS=(evaluation stud*)) OR TS=(controlled trial*)) OR TS=(follow-up stud*)) OR TS=(prospective stud*)) OR TS=(random*)) OR TS=(placebo*)) OR TS=((single blind*))) OR TS=((double blind*)) |
| #4 #1 AND #2 AND #3 |

### Supplementary Table A.4. Search Strategy for Econlit

| #1 TI ( interdisciplinary or multidisciplinary or "cross disciplinary" or "fringe benefit" or "fringe benefits" or "global budget" or "global budgets" or "line item budgets" or transdisciplinary or "patient care team" or "medical care team" or "healthcare team" or MDT or "multiple providers" or interprofessional or "health team" or teamwork ) |
| --- |
| #2 AB ( interdisciplinary or multidisciplinary or "cross disciplinary" or "fringe benefit" or "fringe benefits" or "global budget" or "global budgets" or "line item budgets" or transdisciplinary or "patient care team" or "medical care team" or "healthcare team" or MDT or "multiple providers" or interprofessional or "health team" or teamwork ) |
| #3 #1 OR #2 |
| #4 AB ('crossover procedure' OR 'double-blind procedure' OR 'randomized controlled trial OR 'single-blind procedure' OR comparative study OR clinical trial OR follow-up study) |
| #5 TI ('crossover procedure' OR 'double-blind procedure' OR 'randomized controlled trial OR 'single-blind procedure' OR comparative study OR clinical trial OR follow-up study) |
| #6 #4 OR #5 |
| #7 #3 AND #6 |

### Supplementary Table A.5. Search Strategy for OpenGrey

| (interdisciplinary OR multidisciplinary OR "cross disciplinary" OR "fringe benefit" OR "fringe benefits" OR "global budget"  OR "global budgets" OR "line item budgets" OR transdisciplinary OR "patient care team" OR "medical care team"  OR "healthcare team" OR MDT OR "multiple providers" OR interprofessional OR "health team" OR teamwork)  AND ("clinicaltrial*" OR "research design" OR "comparative stud*" OR "evaluation stud*" OR "controlled trial*"  OR "follow-up stud*" OR "prospective stud*" OR "random*" OR "placebo*" OR "single blind*" OR "double blind*") |
| --- |

### Supplementary Table A.6. Search Strategy for WangFang

| Publication type：Journal article |
| --- |
| #1 主题 "慢性病" OR "慢病" OR "慢性非传染性疾病" OR "慢性疾病" OR "慢非" |
| #2 主题 "多学科" OR "团队" OR "协作" OR "合作" OR "MDT" |
| #3 主题 "随机" OR "安慰剂" OR "对照" OR "盲法" OR "单盲" OR "双盲" OR "三盲" |
| #4 #1 AND #2 AND #3 |

### Supplementary Table A.7. Search Strategy for CNKI

| Publication type：Journal article |
| --- |
| #1 SU% = ( '慢性病' + '慢病' + '慢性非传染性疾病' + '慢非' + '慢性疾病' ) OR AB = ( '慢性病' + '慢病' + '慢性疾病' + '慢性非传染性疾病' + '慢非' ) |
| #2 SU% = ('多学科' + '团队' + '协作' + '合作' + 'MDT') OR AB = ('多学科' + '团队' + '协作' + '合作' + 'MDT') |
| #3 SU% = ('随机' + '安慰剂' + '对照' + '盲法' + '单盲' + '双盲' + '三盲') OR AB = ('随机' + '安慰剂' + '对照' + '盲法' + '双盲' + '三盲') |
| #4 #1 AND #2 AND #3 |

### Supplementary Table A.8. Search results

| **number** | **Data source** | **Search results** |
| --- | --- | --- |
| 1 | PubMed | 624 |
| 2 | Embass | 511 |
| 3 | Web of Science | 1521 |
| 4 | EconLit | 18 |
| 5 | OpenGrey | 125 |
| 6 | CNKI | 322 |
| 7 | WanFang | 340 |
| 8 | Other source | 2 |
| 9 | Total | 3463 |

### Supplementary Fig. A.1. Risk of bias summary


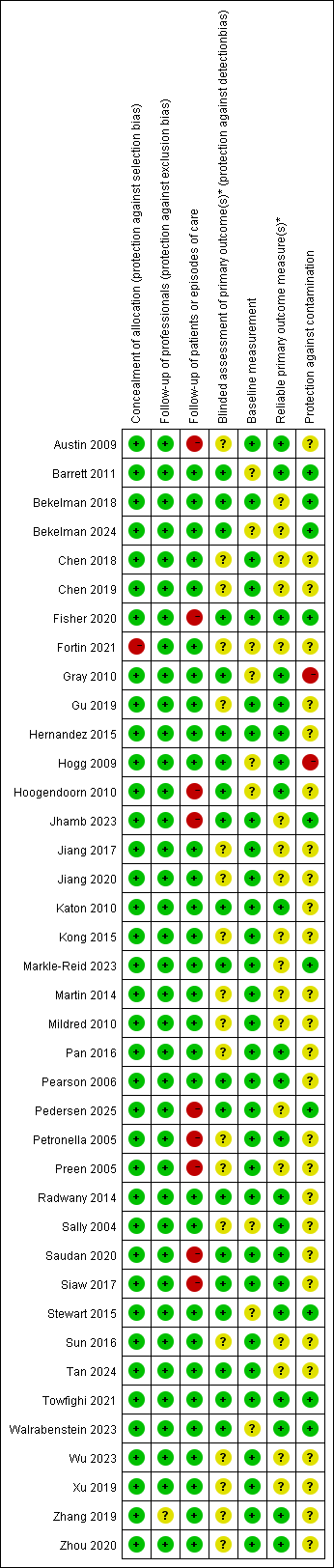

Supplement: Supplementary file 1 — Supplementary Material 1 [file 12875_2025_2814_MOESM1_ESM.docx]
